# Superior Osteo-Inductive and Osteo-Conductive Properties of Trabecular Titanium vs. PEEK Scaffolds on Human Mesenchymal Stem Cells: A Proof of Concept for the Use of Fusion Cages

**DOI:** 10.3390/ijms22052379

**Published:** 2021-02-27

**Authors:** Enrico Ragni, Carlotta Perucca Orfei, Alessandro Bidossi, Elena De Vecchi, Natale Francaviglia, Alberto Romano, Gianluca Maestretti, Fulvio Tartara, Laura de Girolamo

**Affiliations:** 1 Laboratorio di Biotecnologie Applicate all’Ortopedia, IRCCS Istituto Ortopedico Galeazzi, Via R. Galeazzi 4, I-20161 Milano, Italy; enrico.ragni@grupposandonato.it (E.R.); carlotta.perucca@grupposandonato.it (C.P.O.); 2 Laboratory of Clinical Chemistry and Microbiology, IRCCS Istituto Ortopedico Galeazzi, Via R. Galeazzi 4, I-20161 Milano, Italy; alessandro.bidossi@grupposandonato.it (A.B.); elena.devecchi@grupposandonato.it (E.D.V.); 3 Neurochirurgia Funzionale, Istituto Ortopedico Villa Salus, Contrada Spalla, I-96010 Melilli, Italy; francaviglianatale@gmail.com; 4 Unità Operativa di Neurochirurgia, Humanitas Istituto Clinico Catanese, Contrada Cubba Marletta 11, I-95045 Misterbianco, Italy; romanoalb@gmail.com; 5 HFR Fribourg-Université de Fribourg, CH-1708 Fribourg, Switzerland; Gianluca.Maestretti@h-fr.ch; 6 Istituto Clinico Città Studi, I-20131 Milan, Italy; tartarafulvio@gmail.com

**Keywords:** mesenchymal stem cells, osteogenesis, titanium, PEEK, cage, arthrodesis, cervical spine, lumbar spine, osteo-induction, osteo-conduction

## Abstract

Fusion cages composed of titanium and its alloys are emerging as valuable alternative to standard polyetheretherketone (PEEK) ones routinely used in cervical and lumbar spine surgery. Aim of this study was to evaluate osteo-inductive and osteo-conductive ability of an innovative trabecular titanium (T-Ti) scaffold on human mesenchymal stem cells (hMSCs), in both absence and presence of biochemical osteogenic stimuli. Same abilities were assessed on PEEK and standard 2D plastic surface, the latter meant as gold-standard for in vitro differentiation studies. hMSCs adhered and colonized both T-Ti and PEEK scaffolds. In absence of osteogenic factors, T-Ti triggered osteogenic induction of MSCs, as demonstrated by alkaline phosphatase activity and calcium deposition increments, while PEEK and standard 2D did not. Addition of osteogenic stimuli reinforced osteogenic differentiation of hMSCs cultured on T-Ti in a significantly higher manner with respect to standard 2D plastic culture surfaces, whereas PEEK almost completely abolished the process. T-Ti driven differentiation towards osteoblasts was confirmed by gene and marker expression analyses, even in absence of osteogenic stimuli. These results clearly indicate superior in vitro osteo-inductive and osteo-conductive capacity of T-Ti compared to PEEK, and make ground for further studies supporting the use of T-Ti cages to improve bone fusion.

## 1. Introduction

Worldwide, one of the most common medical problems is represented by cervical and lumbar spine disorders. Low back and neck pain were the top and fourth leading causes for years lived with disability between 1990 and 2013 and, overall, back pain was the leading cause in 94 developing and 45 developed countries, as reported by the Global Burden of Disease Study 2013 [1]. To date, current treatments are one of the biggest driving causes in health care expenditures, with the estimated annual cost of spine care in the US being close to $100 billion after a 175% increase from a decade ago [2,3].

Often damage of ligaments, joints and vertebral muscles are a consequence of degenerative changes, leading to greater susceptibility to injuries and chronic pain involving both spinal nerve radices and neural endings into the inflamed and degenerated disc [4]. Although non-operative options should always be preferred [5], several factors has led to an increase in the rate of surgical treatments, valued close to $3 billion in Europe and $10 billion in the US in 2020 [6], among which the rapid evolution of improved surgical techniques [3]. In this scenario, the composition and design of fusion cages still represents an open challenge. As a general rule, an ideal cage has to restore the correct spine alignment and stabilize the motion segment, eventually allowing long-term spine fusion and osteo-integration. To date, two key materials are preferentially used, namely titanium (Ti) and its alloys, and polyetheretherketone (PEEK) [7], both having advantages and disadvantages. PEEK is recognized as a suitable implant material for hard tissue application because it possesses a similar flexural modulus to cortical bone, so it bends and bears weight like the body’s own tissues [8]. This is how PEEK is able to avoid stress shielding, and how the polymer encourages more effective healing in native bone. Also, PEEK has good chemical, thermal and radiation stability [9], and low density (radiolucency), allowing for easier fusion assessment [8]. In fact, PEEK is completely invisible on MRIs, CT scans and X-rays, so surgeons can easily monitor post-operative progress and confirm osteointegration [9]. However, a crucial limitation with PEEK is that it is chemically inert, forming a biofilm layer that inhibits bony growth into the cages from the vertebral end plates, resulting in pseudarthrosis or non-fusion, which has limited its wider application [10]. In this frame, Ti has shown higher ability to support osteo-integration [11]. Consistently, recent reports and literature meta-analyses reported not only a comparable rate of subsidence but a better prevalence of fusion for titanium or titanium-coated cages with respect to nude PEEK [12,13].

These results are presumably due to chemical structure and architecture of PEEK [14]. In this view, highly porous trabecular titanium (T-Ti), with its structure resembling cancellous bone, may assure an even improved ground for bone in-growth and bone cells, as suggested by osteoblasts proliferation and differentiation in vitro [15], bone in-growth in cancellous and cortical bones in vivo [16], and sagittal alignment restoration with deformities correction in patients treated with custom-made T-Ti cages [17]. This is consistent with a recent survey on British surgeons’ practice and implant preferences in lumbar fusion surgery, where titanium was reported as the most frequently used material for fusion implants and porous/trabecular implants largely being the first option [18].

Due to the lack of a direct comparison between PEEK and T-Ti properties on progenitor cells in the same culture conditions, in this study the behavior of human mesenchymal stem cells (hMSCs) seeded onto either PEEK or highly porous T-Ti scaffolds were evaluated and compared. In particular, proliferation rate, acquisition of an osteoblast-like phenotype and production of calcified extracellular matrix in absence (control medium, CM) or presence (osteogenic medium, OM) of biochemical osteogenic stimuli were evaluated. These features were also assessed on the standard 2D plastic surface used in laboratory practice, a not clinically relevant material but a gold-standard for in vitro differentiation studies, used herein as a positive control for induction of osteogenic commitment. To provide a complete observation of the phenomena, several techniques including confocal and scanning electron microscopy (SEM), X-ray spectroscopy, biochemical assays, gene expression and protein analyses were sifted. 

## 2. Results

### 2.1. hMSCs Characterization

**Figure 1:**
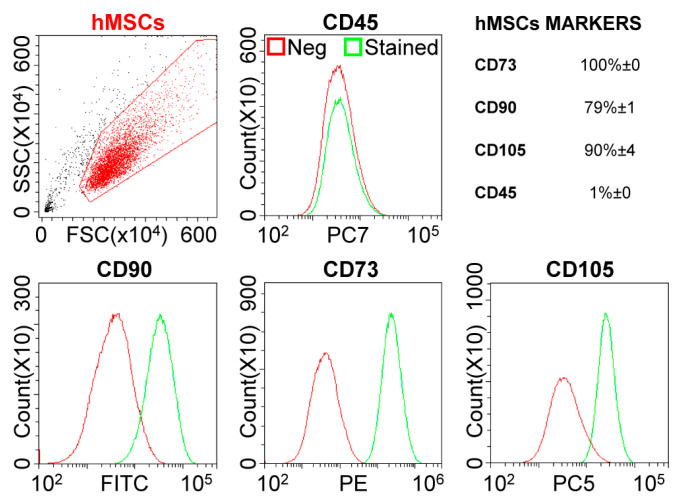
hMSCs phenotype characterization. Flow cytometry analysis of negative (CD45) and positive MSC (CD73, CD90 and CD105) mesenchymal markers staining, confirming hMSCs identity. Representative plots are shown. For positivity values, mean of three independent donors is presented.

The immunophenotypical characterization of hMSCs confirmed their biological signature, with a strong positivity for mesenchymal epitopes (CD73-CD90-CD105) and absence of haematological marker CD45 (Figure 1).

### 2.2. hMSC Morphology and Adhesion onto T-Ti and PEEK

**Figure 2:**
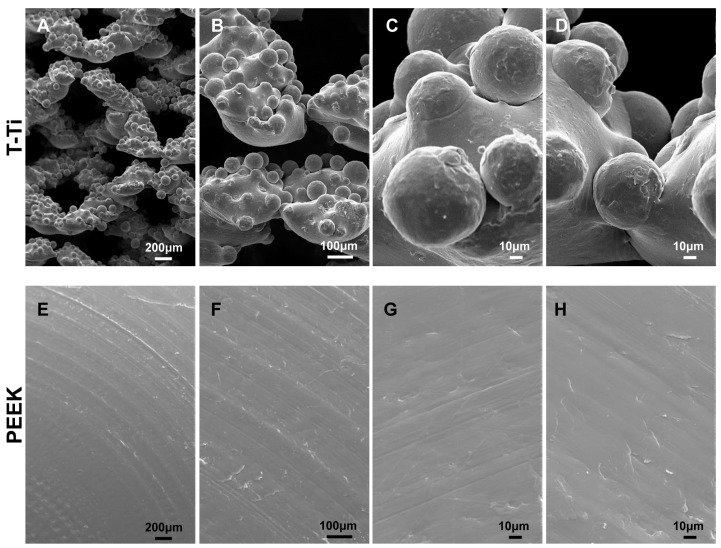
Scaffolds structure. SEM images of nude T-Ti (**A**–**D**) and PEEK (**E**–**H**) scaffolds before hMSCs seeding. T-Ti scaffold had a regular trabecular structure while PEEK scaffold showed a smooth surface. Magnification: A/E 35×, B/F 100×, C/D/G/H 500×.

**Figure 3:**
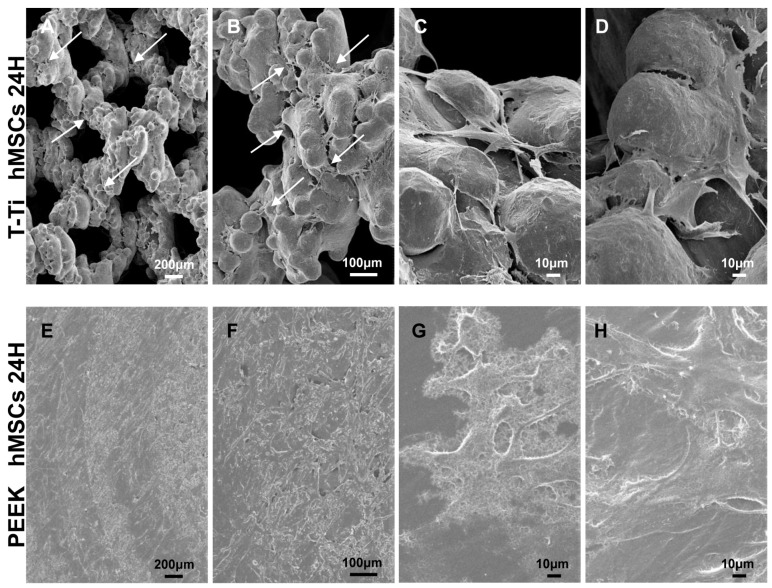
hMSCs morpholgy 24 h after seeding on T-Ti and PEEK scaffolds in CM by SEM. (**A**–**D**) hMSCs seeded on T-Ti scaffold showed a spread morphology, regular borders and the formation of filopodia (white arrows in lower magnifications) both connecting adiacent cells and bridging pores. (**E**–**H**) hMSCs seeded on PEEK scaffold showed a less regular morphology with jagged borders. Magnification: A/E 35×, B/F 100×, C/D/G/H 500×.

**Figure 4:**
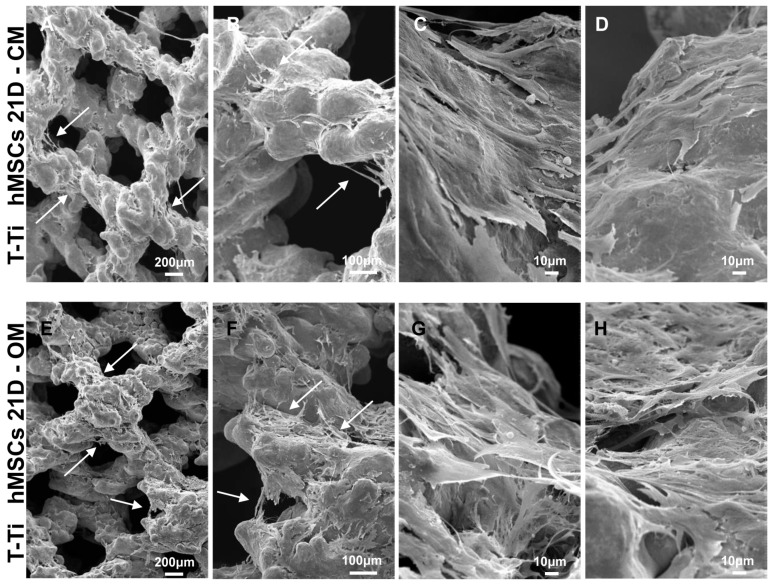
hMSCs morphology 21 days after seeding on T-Ti scaffolds in CM and OM by SEM. (**A**–**D**) hMSCs in CM showed complete colonization of the scaffold and bridges across pores (white arrows), with formation of rigid lamellar structures due to extracellular matrix. (**E**–**H**) hMSCs in OM showed complete colonization of the scaffold with several briding structures (white arrows), and the presence of rigid lamellar structures with a more complex and interconnected topology. Magnification: A/E 35×, B/F 100×, C/D/G/H 500×.

**Figure 5:**
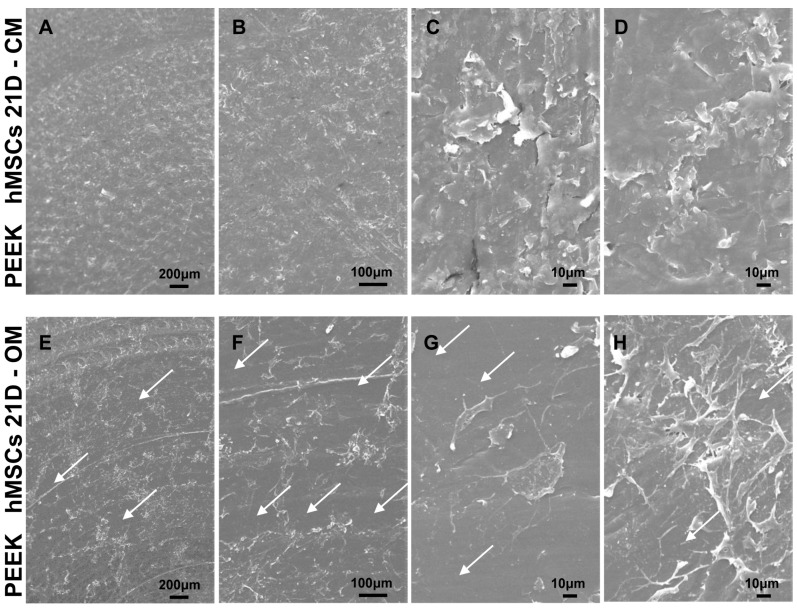
hMSCs morphology 21 days after seeding on PEEK scaffolds in CM and OM by SEM. (**A**–**D**) hMSCs in CM showed complete colonization of the scaffold. (**E**–**H**) hMSCs in OM showed irregular organization with several empty spaces (white arrows). Magnification: A/E 35×, B/F 100×, C/D/G/H 500×.

**Figure 6:**
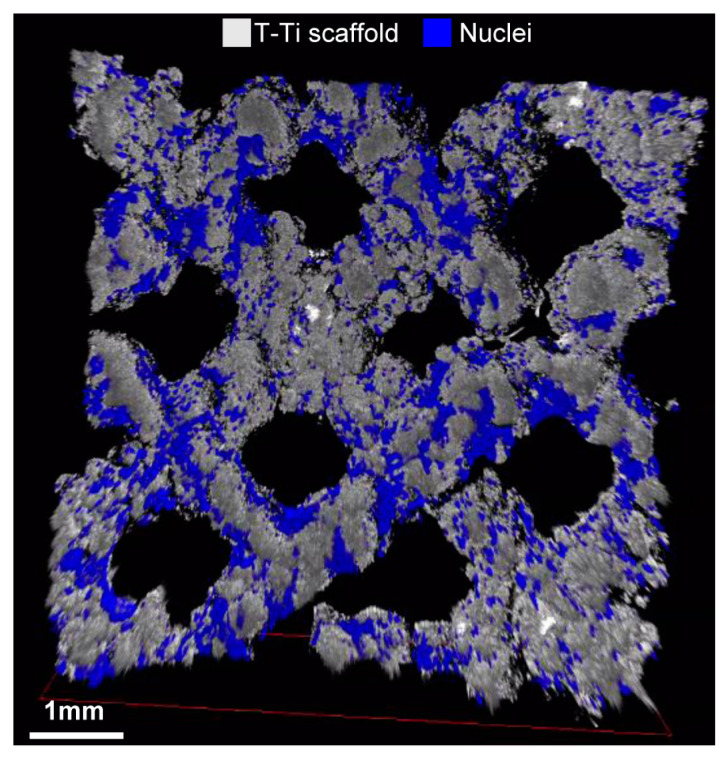
T-Ti colonization in OM 21 days after seeding assessed by confocal microscopy. hMSCs, visualized in blue trhough DAPI staining of the nuclei, homogenously colonized the T-Ti scaffold, in grey. This image is a frame of a confocal microscopy acquired video presented in Supplementary File S1.

Empty T-Ti scaffold showed a trabecular surface with a regular three-dimensional porous structure (Figure 2A–D), while empty PEEK scaffold showed a smooth bi-dimensional surface (Figure 2E–H). One day after seeding in CM, hMSCs on T-Ti scaffold showed a spread morphology and the initial formation of filopodia, with cellular processes forming bridges across porous surface and indicating the biocompatibility of T-Ti (Figure 3A–D). On PEEK scaffold, cells were visible although with more contracted and irregular shape (Figure 3E–H). After 21 days, in both CM and OM hMSCs completely colonized the T-Ti scaffold acquiring a “squared” shape with several bridging structures along scaffold lobes and pores, more frequent and complex in OM (Figure 4A–H). In both culture conditions, abundant and lamellar extra cellular matrix (ECM) with a homogeneous and well organized appearance was detectable, although more rigid and interconnected in OM. Differently, after 21 days, hMSCs cultured on PEEK in CM resulted tightly packed and coverying the entire scaffold surface, while in OM appeared more spread and irregularly organized with visible empty spaces, suggesting a reduction in cellularity (Figure 5A–H). Confocal microscopy of T-Ti scaffold after 21 days in OM confirmed the homogenous colonization of the scaffold (Figure 6 and Supplementary File S1).

### 2.3. hMSCs Proliferation 

**Figure 7:**
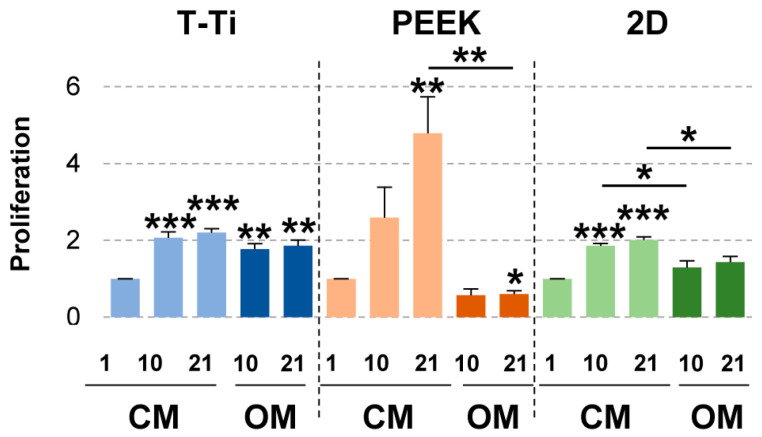
Biochemical analysis of hMSCs proliferation on PEEK, T-Ti and 2D surfaces. Values are calculated with respect to day 1–CM condition, set as 1, for each data group. Values are reported as mean ± SEM. N = 3. Asterisks on top of columns indicate significant *p*-values with respect to day 1–CM condition for each material; asterisks on top of lines indicate significant *p*-values between columns at the edges of each line. * for *p*-value ≤ 0.05, ** for *p*-value ≤ 0.01, *** for *p*-value ≤ 0.001.

hMSCs cultured in CM grew progressively during the three weeks of culture on PEEK, while on T-Ti the cell growth at day 10 and 21 was comparable (Figure 7). In OM, hMSCs cultured on T-Ti showed a similar growth trend. Conversely, hMSC maintained in OM on PEEK showed a reduction in cell number that became significant at 21 days (*p*-value ≤ 0.05), confirming the lacunae observed by SEM, together with a relevant viability reduction up to final 62% (*p*-value = 0.096 at day 21). Viability did not significanlty change in the other experimental conditions (data not shown). In standard 2D cell culture, proliferation was observed only in CM, with values very similar to T-Ti and comparable at 10 and 21 days.

### 2.4. Osteogenic Markers

#### 2.4.1. Alkaline Phosphatase Activity (ALP)

**Figure 8:**
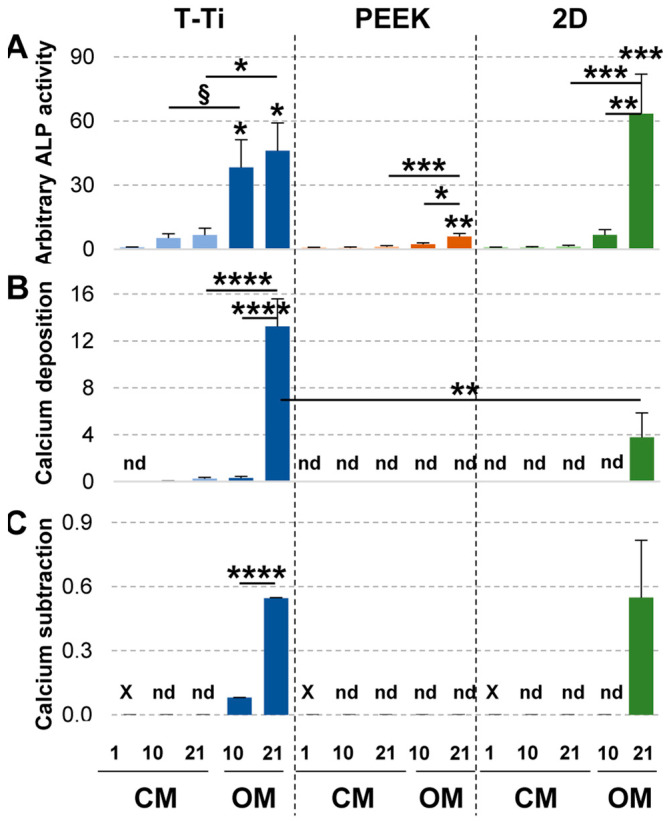
Biochemical analyses of hMSCs ALP activity and Calcium metabolism on PEEK, T-Ti and 2D surfaces. (**A**) Cellular ALP activity on the different surfaces under study. Arbitrary ALP values per 1000 cells are presented, setting as 1 the value of day 1–CM condition on 2D cell culture surface. (**B**) Calcium deposition, in µg from 1000 cells, in either control or osteogenic media. Absolute values are presented. *nd* stands for for not detected. (**C**) Calcium subtraction, in µg, from culture media, either control or osteogenic, from 1000 hMSCs in 96 h after the last medium change. Absolute values are presented. *X* stands for not assayed. *nd* stands for for not detected. Values are reported as mean ± SEM. N = 3. Asterisks on top of columns indicate significant *p*-values with respect to day 1–CM condition for each material; asterisks on top of lines indicate significant *p*-values between columns at the edges of each line. § for *p*-value ≤ 0.1, * for *p*-value ≤ 0.05, ** for *p*-value ≤ 0.01, *** for *p*-value ≤ 0.001, **** for *p*-value ≤ 0.0001.

At beginning of the treatments, hMSCs on the 3 materials showed a comparable arbitrary ALP activity (Figure 8A). In CM cellular ALP did not increase on both PEEK and cell culture plates (Figure 8A). On T-Ti, albeit without biochemical osteogenic stimuli, ALP increased already at 10 days remaining stable until 21 days (*p*-value ≤ 0.1), with values similar to that of the gold-standard cell culture plates observed at 10 days in OM. The osteogenic condition further increased the ALP production by hMSCs seeded on T-Ti scaffold, that showed the highest ALP increase at 10 days of the all data set, even when compared with standard 2D cultures. At 21 days, standard 2D cultures reached the more pronounced increment, albeit not statistically significant with respect to T-Ti that showed a further increase with respect to 10 days. On PEEK, even in OM, ALP increase was weak, with the 21 days’ value comparable to T-Ti in CM.

#### 2.4.2. Calcium Deposition and Subtraction to Culture Medium 

In CM, calcium deposition was not detected for hMSCs grown on PEEK or cell culture plates (Figure 8B). Conversely, hMSCs grown on T-Ti showed a constant calcium increase, with the 21 days’ value comparable to that of cell grown on T-Ti in OM for 10 days. Notably, at 21 days in OM, T-Ti resulted in a calcium deposition higher than in cell culture plates. PEEK did not stimulate calcium deposition even in OM.

The calcium subtraction from media was also assessed as a confirmation of the potential osteogenic effect of the different surfaces and/or media. In CM, no reduction in supernatants calcium levels was observed (Figure 8C). In OM calcium subtraction was observed for hMSCs grown on T-Ti scaffold at both time points, with significant increment at day 21 (*p*-value ≤ 0.0001). At 21 days of treatment on 2D cell culture plates calcium reduction was comparable to the values observed at the same time point on T-Ti surface, whereas no effect was detected at 10 days. Interestingly, no reduction for PEEK scaffold at both time points was observed in osteogenic conditions.

**Figure 9:**
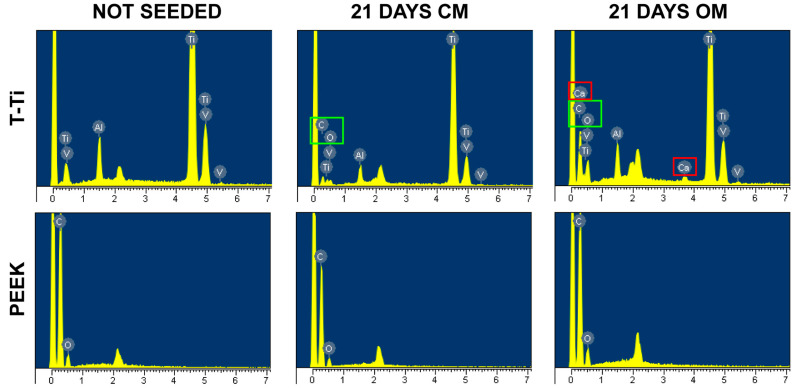
EDS analysis of elements on nude and 21 days hMSCs-cultured PEEK and T-Ti scaffolds. T-Ti scaffold showed the presence of its composing elements, such as Titanium (T), Aluminium (A) and Vanadium (V), while PEEK scaffold Carbon (C) and Oxygen (O). Twenty-one days after hMSCs seeding, on T-Ti scaffolf both Carbon and Oxygen, due to cellular structures, were detcted in both CM and OM (C and O, boxed in green), while Calcium (Ca, boxed in red) only in OM. On PEEK, no Calcium was detected in both culture conditions. X-axis values are in keV.

The presence of calcium deposits was also assessed by X-ray spectroscopy on PEEK and T-Ti scaffolds cultured in both CM and OM for 21 days. The analysis showed the presence of calcium on the T-Ti constructs cultured in OM, consistently with the very high values detected in the biochemical assay that allowed to exceed the spectroscopy sensitivity limit given by the small area assayed (Figure 9).

### 2.5. Gene and Protein Expression on T-Ti Scaffold

**Figure 10:**
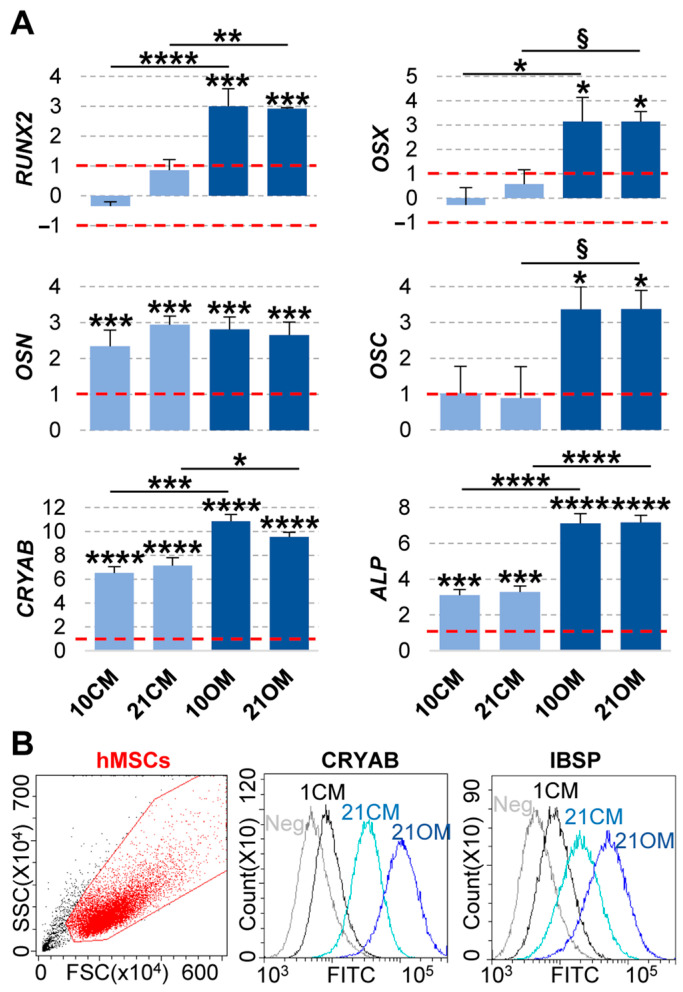
Gene and protein expression analyses at 21 days on T-Ti in CM and OM. (**A**) Gene expression analysis of osteogenic markers *RUNX2*, *OSX*, *OSN*, *OSC*, *CRYAB* and *ALP* 21 days after seeding on T-Ti scaffold in both CM (shown in light blue) and OM (dark blue). Values are reported as Log_2_(FC) with respect to time day 1 in CM (24 h after seeding). Dotted red lines set as ± 1 for Log_2_(FC) threshold. Values are reported as mean ± SEM. N = 3. Values are reported as mean ± SEM. N = 3. Asterisks on top of columns indicate significant *p*-values with respect to day 1–CM condition for each material; asterisks on top of lines indicate significant *p*-values between columns at the edges of each line. § for *p*-value ≤ 0.1, * for *p*-value ≤ 0.05, ** for *p*-value ≤ 0.01, *** for *p*-value ≤ 0.001, **** for *p*-value ≤ 0.0001. (**B**) Flow cytometry analysis for CRYAB and IBSP in hMSCs at time day 1 in CM and 21 days in both CM and OM. Neg plot stands for cells stained only with secondary antibody, and only time day 1 in CM is shown for clarity.

To confirm the osteo-conductive and osteo-inductive potential of T-Ti suggested by the previous experiments, mRNA levels of *RUNX2* and *OSX* transcription factors, *OSN* glycoprotein, *OSC* hormon, *CRYAB* chaperon and *ALP* were assayed by qRT-PCR. In OM, all osteo-related markers resulted significantly and strongly upregulated with comparable values at 10 and 21 days, confirming the prompt and durable capacity of T-Ti scaffold to support differentiation in osteogenic conditions (Figure 10A). In CM, the response was present but at lower levels. *RUNX2* and *OSX* transcription factors showed a trend towards upregulation at 21 days, with *RUNX2* close to significance for fold change (Log_2_(FC) = 0.9). *OSN*, *CRYAB* and *ALP*, significantly increased their transcript levels at both 10 and 21 days (Figure 10A). Comparing CM and OM, *ALP* mRNA differential increase corroborated the ratio observed in the biochemical assays for the enzymatic activity. The same confirmation of mRNA values at protein level was observed by flow cytometry for CRYAB (Figure 10B). This technique also confirmed the induction on T-Ti in both culture conditions of the osteogenic marker IBSP, with OM again stimulating a higher level of this protein. Overall, qRT-PCR and flow cytometry data substantiated biochemical data about osteogenic commitment, although at different levels, of hMSCs on T-Ti surfaces in both control and osteogenic conditions.

## 3. Discussion

The present set of experiments directly compared the in vitro hMSCs osteogenic commitment when seeded on PEEK or T-Ti scaffolds in the same culture conditions. The study findings showed the osteo-inductive and osteo-conductive capacity of T-Ti scaffolds, by means of different techniques encompassing biochemical and molecular assays. SEM also showed that hMSCs are able to homogenously colonize T-Ti scaffolds. Conversely, PEEK surfaces supported proliferation without osteogenic stimuli, but resulted unable to both induce and even conduce osteogenic commitment.

Historically, PEEK has been described as an optimal solution for cages due to its high modulus of elasticity, close to cortical bone [19], and its radiolucency allowing for easier fusion assessment [8]. Nevertheless, the latter feature, may have led to an overestimation of the PEEK performance. In fact, recently, in patients treated for interbody fusion with PEEK cages, unfavorable CT scan results have been described [14,20]. This may be due to the increment in magnetic resonance instrumentation performances and use, since conventional plain X-ray evaluation may identify more easily interbody fusion events given PEEK radiolucency [14]. Observed suboptimal fusion rates might depend on PEEK lower stiffness and its hydrophobic characteristics, leading to insufficient initial stability and poor background for bony bridging and solid fusion. The chemical structure, that is inert and with low osteogenic properties [14], might also have an influence on bone cells and consequent bone ingrowth. Our results on PEEK scaffold showed that hMSCs may adhere and proliferate in CM, a culture condition pretty far from the in vivo biochemical environment of a growing/repairing bone, albeit with complete absence of osteo-inductive properties. Moreover, SEM showed that hMSCs adhere to PEEK with an irregular and contracted morphology that is retained also after 21 days in both OM and CM, regardless positive proliferation. Observed morphology is quite different from what displayed under conventional 2D surfaces [21], where cellular extensions and pseudopods arising from each cell and extending through each other have been reported, suggesting the contribution of the material, together with the surface architecture, to altered morphology. Notably, in OM, more resembling the in vivo presence of factors able to support bone cells and bone growth, hMSCs cultured on PEEK not only showed absence of proliferation, but a reduction in number and viability and a very poor response in terms of osteogenic commitment. The latter resulted greatly lower than in ordinary 2D cell culture plastic, suggesting lack of osteo-conductive capacity and possibly anti-osteogenic properties. Therefore, these results might explain on a cellular level the poor PEEK cages outcomes in terms of bone bridging and fusion that have been observed with more sophisticated imaging techniques in patients, or after scaffold explant from long bones in vivo [22]. 

To overcome the poor integration of smooth PEEK with adjacent bone upon implantation, modification of its surface by incorporating trabecular microstructures has been investigated and proven to be effective on bone cells [23,24]. Although promising, some technical issues have still to be overcome to allow large-scale 3D PEEK diffusion on the market. As an example, traditional manufacturing techniques such as porogen leaching are restricted to limited areas when creating porous structures on cages [25]. Additive manufacturing, including 3D printing, has sparked cage manufacturers’ interest and consists of a wide range of different technologies which are dependent on the specific application [26]. Nevertheless, AM of PEEK is challenging due to its high melting temperature and thermal gradients that are related to interlayer bonding strength [27], crystallinity [28] and deformation [29]. Consistently, during 3D printing, temperature together with speed and layer thickness might significantly affect bonding intensity between adjacent filaments ultimately altering cages’ mechanical strength [30]. Thus, overall parametric optimization of the 3D printing process will be crucial to improve this approach. Alongside, to obtain a porous architecture on PEEK scaffolds, surface casting has also been explored. The most used technique is plasma-spraying to coat PEEK with roughened titanium or hydroxyapatite [31]. These implants have shown high levels of osteo-integration [32,33]. However, the coating, even if relatively thin (typically 100–200 μm), may obscure visualization of the bone-implant interface and coating wear or delamination were observed with particle-induced osteolysis and aseptic loosening [34,35,36]. Thus, the idea of porous structures in order to maximize the bone ingrowth on prosthesis surfaces, without the need of specific skillset or durability issues, laid the foundation for the cages in whole titanium, combining its innate ability to support osteo-integration [11] and easiness of innovative and cost-effective 3D printing technologies (e.g., electron beam melting technology in this study) [37]. T-Ti has a porous and rough structure resembling a trabecular spongy bone and allowing cells to have access to nutrients and space to grow within pores. On a biological level, this may favor superior bone and bone cells ingrowth leading to a bridge with the implant and an enhanced biomechanical compatibility [38]. Consistently, in vitro, the structural, chemical and physical features of T-Ti allowed the adhesion and proliferation of bone and endothelial progenitors, suggesting also vascularization and thus favoring osteo-integration [15,39,40,41,42,43,44]. As a consequence, in vivo, the presence and size of pores in titanium implants showed a consistent positive effect on the amount of new bone growth and integration [45].

In this study, hMSCs were able to adhere to T-Ti scaffolds, showing the presence of filopodia and spreading along the metal surface and within pores with tiny cellular processes, an indicator of the scaffold biocompatibility. After 21 days, in both CM and OM cells changed their morphology, partially losing the typical “fibroblast-like” morphology of mesenchymal cells and progressively acquiring a more “squared” shape, typical of osteoblasts. Differently than 2D cultures, T-Ti promoted proliferation also under osteogenic stimuli, consistently with other reports on hMSCs seeded on different 3D scaffolds/materials [46,47]. This is of importance since implant colonization relies on a multistep process that includes cell adhesion to the surface of a material, followed by proliferation and differentiation, and involves the production of specific proteins and the deposition of calcium phosphate in the ECM [48]. Consistently, after colonization of the scaffolds, hMSCs started to deposit calcium-rich ECM. With respect to 2D surfaces, as PEEK and traditional cell culture plates, T-Ti was able to induce cellular ALP activity in CM. This is consistent with previous reports showing that cells grown on a rough surface produced bigger amount of ALP even in absence of osteogenic stimuli [49]. ALP regulates organic and inorganic metabolism via the hydrolyzation of phosphate esters, and its role as a marker for osteogenic activity has been consistently solidified [50]. Further, calcium deposition, another marker of effective hMSCs differentiation towards osteogenic phenotype [51], was detected at 10 and more consistently at 21 days and confirmed the presence of ECM calcification. Therefore, the induction of ALP and calcium deposition in absence of osteogenic stimuli, and compared to inert materials as PEEK or standard cell culture plates, indicated the innate osteo-inductive capability of the T-Ti scaffold. Moreover, its osteo-conductive properties emerged as well. In fact, in OM at 10 days, ALP levels increased more rapidly and resulted higher than in 2D cell culture plates, and not statistically different at 21 days. Concerning calcium, both subtraction from medium and deposition were detected already at 10 days and absent in standard 2D cell culture plates, with deposition that resulted significantly more elevated also at 21 days. These results suggest that T-Ti scaffold is able to quickly boost differentiation in osteogenic conditions, and therefore “conduce” osteogenesis in an environment resembling growing/repairing bone.

Osteogenic commitment on T-Ti scaffolds was also confirmed by qRT-PCR. Under osteogenic conditions, all the assayed markers resulted clearly and significantly upregulated, with similar levels at 10 and 21 days, again indicating the early and sustained phenotype change. In CM, *RUNX2*, the early and master regulator of osteogenic differentiation [52], even in absence of osteogenic stimuli had a trend towards expression increment, with Log_2_(FC) = 0.9 at 21 days with respect to Log_2_(FC) of 3.0 and 2.9 in OM at 10 and 21 days, respectively. In absence of chemical stimuli, as previously shown [53,54], *RUNX2* increment might be due to cell mechanical stimulation by bending or stretching of the ECM in the 3D environment, as observed by SEM with hMSCs engulfing and embracing T-Ti lobes, that can directly control cell behavior, including adhesion and differentiation [55]. Notably, other early/mid markers as *ALP*, confirming biochemical data, and *CRYAB*, whose upregulation significantly enhances osteogenic differentiation through the canonical Wnt/β-catenin signaling [53], resulted strongly increased, albeit again at a lower extent with respect to OM condition. *CRYAB* mRNA differential increment between CM and OM was confirmed by flow cytometry, similarly to IBSP, another early/mid marker connected with bone ECM deposition [52]. Osteonectin levels, usually found in young osteocytes and marker of the osteoblastic functional differentiation [56], resulted similarly upregulated in CM and OM. Eventually, the late osteoblast marker Osteocalcin [52] showed a weak upregulation in CM, albeit not significant, consistently with low levels of its regulator *RUNX2* [57]. Overall, qRT-PCR data confirmed osteogenic commitment of hMSCs in both CM and OM, although more pronounced in OM, and that the trabecular structure of the T-Ti scaffold, even in absence of external stimuli, drives the osteogenic differentiation of hMSCs.

In the clinical practice, in agreement with the results herein presented, British and Dutch surgeons reported that porous/trabecular implants together with nonporous materials with rough/threaded topology were the first option (96.7%), while implants with smooth surface were consistently disregarded [18,58]. Also, and again confirming our data, the preferred material for fusion implants was titanium (54.3%), largely more addressed than PEEK (14.7%) and tantalum (28.6%). Similar to titanium, also trabecular tantalum cages have been used in clinical practice for many years given the interesting osteogenic properties and osteointegration capacity [59], but so far no further investments have been done in the development of more innovative cages in terms of size, morphology and technical options (lateral approach). Therefore at the moment 3D printed porous titanium represents the material of greatest perspective and studies aimed at greater understanding of its biological potential can be significant. Future studies directly comparing well characterized *vs* new players, as trabecular Titanium *vs* highly porous PEEK, will be necessary.

In conclusion, this study demonstrated in vitro that trabecular titanium is a biocompatible surface for hMSCs adhesion, proliferation and differentiation towards the osteogenic lineage, even without the addition of osteogenic factors. Along with its osteo-inductive capacity, T-Ti scaffold enhanced differentiation under osteogenic stimuli, being therefore osteo-conductive and, overall, a suitable material to promote osteo-integration and fusion. On the contrary, in the same experimental environment, PEEK showed lack of osteo-induction and even poorer response under osteogenic stimuli. Future in vivo studies and trials will be needed to confirm T-Ti as a superior option for cages in terms of fusion and biocompatibility.

## 4. Materials and Methods

### 4.1. Ethics Statement

The research was conducted at IRCCS Istituto Ortopedico Galeazzi under Institutional Review Board approval (San Raffaele Hospital Ethics Committee approval in date 8 March 2018, registered under number 6/int/2018). Specimens were collected after obtainment of patient informed consent (CI_REGAIN_adulto_v2) and following the 1964 Helsinki declaration and its later amendments.

### 4.2. Human Mesenchymal Stem Cells (hMSCs) Isolation and Expansion

Waste adipose tissue was collected from three healthy female donors (50 ± 7 yo) undergoing liposuction and processed as already reported [60]. Briefly, tissues were digested in type I collagenase (0.075% *w*/*v*, 37 °C, 30 min) (Worthington Biochemical Co, Lakewood, NJ, USA) and filtered with a 100 μm cell strainer. After centrifugation (376× *g*, 5 min), pellets were suspended in DMEM + 10% FBS (GE Healthcare, Piscataway, NJ, USA) and, after count, cells seeded at 5 × 10^3^/cm^2^. Cells were cultured at 37 °C, 5% CO_2_ and 95% humidity and, at passage 5, analyzed by flow cytometry or seeded on the different surfaces. 

### 4.3. hMSCs Immunophenotype Characterization

hMSCs were incubated for 30 min at 4 °C in the dark with anti-human antibodies: CD90-FITC, CD73-PE, CD105-PerCP-Vio700, CD45-PE-Vio770 (Miltenyi Biotec, Bergisch Gladbach, Germany). Unstained samples were used as negative controls, and data were acquired with a CytoFLEX flow cytometer (Beckman Coulter, CA, USA) collecting a minimum of 30,000 events.

### 4.4. Titanium and PEEK Scaffolds

The scaffolds were kindly provided by MT Ortho company (MT Ortho s.r.l., Aci Sant’Antonio, CT, Italy). The trabecular titanium structures, consisting of repeated rhombic dodecahedron unit cells, were manufactured using the Additive Manufacturing technology called Electron Beam Melting, a powder-based bed process, which uses an electron beam to melt a metal powder deposited on the base plate layer by-layer. A fine powder Arcam Ti6Al4V ELI (Grade 23) was used for the production of rhombic dodecahedron micro-lattices. The particle size distribution is between 45 and 100 μm and average diameter of pores in the range of 0.55–0.70 mm. All the lattices for the mechanical tests were designed using Materialise Magics^®^ software. PEEK scaffolds were produced by the Invibio ltd (Victrex, Lancashire, UK) and based on the formula (-C_6_H_4_-O-C_6_H_4_-O-C_6_H_4_-CO-)_n_. The resulting PEEK-OPTIMA^®^ LT1 has the following physical and mechanical properties: density 1300 kgm/cm^3^, Young module 4.1 GPa, tensile strength 99 Mpa, tensile elongation 19%, flexural strength 170 MPa, Relative Thermal Index 260 °C, fusion temperature 337 °C, crystallization temperature 290 °C, glass transition temperature 143 °C. Scaffolds’ size was: 1.7 cm × 1.7 cm × 0.1 cm (side × side × height, squared shape, T-Ti) and 1.7 cm × 0.1 cm (diameter × height, round shape, PEEK).

### 4.5. hMSCs Seeding

Both T-Ti and PEEK scaffolds were placed and maintained in wells of a 12-wells plate. hMSCs were seeded at 1.5 × 10^4^/cm^2^ on 2D surfaces (PEEK scaffolds and standard 12-wells cell culture plates) or at 5 × 10^4^/cm^2^ on 3D T-Ti scaffolds. Every experimental condition has been performed in duplicate for each donor. The difference in seeding densities between 2D and 3D was ruled by preliminary experiments aimed at identifying the cell concentration able to obtain an approximate 50% confluence and surface coverture, due to the three-dimensional trabecular architecture of Titanium that did not allow a sharp calculation of its cell adhesion area. For all determinations, 24 h after seeding, both T-Ti and PEEK scaffolds were moved from the original wells to new wells to avoid the presence of residual cells on the plastic surface below.

### 4.6. hMSCs Culture and Osteogenic Induction

Twenty-four hours after seeding, half samples were maintained in control medium–CM (DMEM + 10% FBS) and half samples were moved to osteogenic medium-OM (MesenCult™ Osteogenic Differentiation Kit, Stemcell Technologies, Cambridge, MA, USA). hMSCs were maintained for 21 days at 37 °C, 5% CO_2_ and 95% humidity, and medium replaced twice a week. At time 1 (24 h after seeding, Time 1 CM), 10 and 21 days (Times 10 CM/OM, control or osteogenic, and 21 CM/OM) hMSCs and scaffolds were analyzed.

### 4.7. SEM and EDS Analyses

T-Ti and PEEK scaffolds, with and without hMSCs at 1 and 21 days, were washed with PBS and fixed for 1 h with 2% glutaraldehyde in 0.1 M Sodium Cacodylate Buffer pH 7.2. Then the scaffolds were washed with Sodium Cacodylate buffer for 30 min, post-fixed with 2% Osmium tetroxide and dehydrated with graded ethanol series, starting with 50, 70, 90 and 100%. After samples were attached onto standard SEM stubs and coated with a thin film of evaporated gold, they were placed in a Zeiss LEO 1430 SEM (Carl Zeiss Jena GmbH, Jena, Germany) coupled with an Oxford detector (Oxford Instruments, Abingdon, UK) for EDS analysis. SEM images were taken from different areas of a sample with varying magnifications (15–1000×). Operating conditions were: accelerating voltage 10–20 kV, probe current 80 µA, and working distance varying from 10.0 to 15.0 mm. Subsequently, scaffolds were inspected for elemental analysis with EDS using the Oxford Instruments INCA ver. 4.04 software.

### 4.8. hMSCs Adhesion and Proliferation Assays

hMSCs adhesion and proliferation was measured with the cell counting kit-8 (CCK-8) cellular proliferation assay (Sigma-Aldrich, St. Louis, MO, USA). At each time point, following manufacturer’s protocol, old supernatant was replaced with new medium, either control or osteogenic, and CCK-8 solution was administered for 2 h, followed by absorbance measurement of the cell supernatant at 450 nm using a Victor X3 microplate reader (PerkinElmer Life and Analytical Sciences, Shelton, CT, USA). A calibration curve was also prepared, associating CCK-8 absorbance readouts with those obtained with pre-determined cell numbers, counted with an automated cell and viability counter (NucleoCounter^®^ NC-3000™, ChemoMetec, Allerod, Denmark) on independent samples plated on 2D plastic surfaces.

### 4.9. hMSCs Cell Viability Assay

hMSCs viability was measured with the CellTiter-Blue^®^ Cell Viability Assay (Promega, Madison, WI, USA). At each time point, following manufacturer’s protocol, old supernatant was replaced with new medium, either control or osteogenic, and CellTiter-Blue solution was administered for 4 h, followed by fluorescence (579Ex/584Em) measurement of the cell supernatant using a Victor X3 microplate reader. A calibration curve associating the cell viability, generated with NucleoCounter^®^ NC-3000™ on independent samples on 2D plastic surfaces, to CellTiter-Blue fluorescence values was also prepared. Cell number obtained with CCK-8 assay was used to normalize fluorescence values.

### 4.10. Cellular Alkaline Phosphatase Activity Assay

At each time point, the culture supernatant was removed and samples were rinsed three times in PBS and incubated for 2 h with 1 mg/mL 4-nitrophenol phosphate in 0.1 M glycine buffer containing 1 mM MgCl_2_, 1 mM ZnCl_2_, pH 10.4 (all reagents from Sigma Aldrich). The absorbance was read at 405 nm on a Victor X3 microplate reader. Cell number obtained with CCK-8 assay was used to normalize absorbance values.

### 4.11. Calcium Deposition Assay

Calcium deposition on T-Ti and PEEK scaffolds and 2D standard plastic surfaces was measured with Calcium Assay Kit (Cosmo Bio Co, Ltd., Tokyo, Japan). Kit internal calibration curve was used to associate absorbance, read at 570 nm on a Victor X3 microplate reader, with Calcium levels. Manufacturer’s protocol was used, with the exception of trichloroacetic acid Calcium release performed for 6 h at RT. Cell number obtained with CCK-8 assay was used to normalize detected Calcium values.

### 4.12. Calcium Subtraction from Culture Supernatants Assay

Calcium subtracted from cell culture supernatants, either control or osteogenic, was measured after 4 days from the last medium replacement, at 10 and 21 days after seeding, with the Colorimetric Calcium Detection Kit (abcam, Cambridge, MA, USA), using fresh control and osteogenic media as comparison and kit internal calibration curve to associate absorbance, read at 570 nm on a Victor X3 microplate reader, with Calcium levels. Manufacturer’s protocol was used. Cell number obtained with CCK-8 assay was used to normalize subtracted Calcium values.

### 4.13. RNA Isolation and Quantitative Real-Time PCR (qRT-PCR)

T-Ti scaffolds at the different time points were washed three times in PBS and hMSCs dissolved in Trizol reagent (Sigma Aldrich). RNA was extracted following manufacturer’s protocol. cDNA was generated using iScript™ gDNA Clear cDNA Synthesis Kit (Bio-Rad, Hercules, CA, USA) following manufacturer’s instructions. qRT-PCR was performed with iTaq Universal SYBR Green Supermix (Bio-Rad) following manufacturer’s protocol and 1 ng/µL cDNA concentration. *RUNX2*, *OSX*, *OSN*, *OSC*, *CRYAB* and *ALP* primer pair sequences will be provided upon request. Expression data were normalized using the housekeeping gene *ACTB* and values reported as log_2_(Fold Change) versus expression on scaffold at time 1 CM (24 h after seeding).

### 4.14. IBSP and CRYAB Protein Detection by Flow Cytometry

T-Ti scaffolds were washed three times in PBS and trypsin used to dissociate hMSCs. Cells were fixed and permeabilized and stained at 4 °C for 30 min with rabbit primary unconjugated antibodies against human Bone Sialoprotein and human Alpha B Crystallin. After two washes in ice-cold FACS buffer (PBS, 2% FBS, 0.1% NaN_3_), cells were stained with Anti-Rabbit IgG H&L (Alexa Fluor^®^ 488) (all antibodies from abcam) at 4 °C for 30 min in the dark. After a final wash, data were acquired with a CytoFLEX flow cytometer (Beckman Coulter), collecting a minimum of 30,000 events and using Anti-Rabbit IgG H&L stained samples as negative controls.

### 4.15. Confocal Microscopy

T-Ti scaffolds were washed in PBS and fixed in 4% paraformaldehyde at 4 °C for 30 min. After 3 washes in PBS, DAPI staining was performed for 10 min at RT. Images of the scaffold were then acquired by means of confocal laser scan microscopy with an upright TCS SP8 (Leica Microsystems CMS GmbH, Mannheim, Germany) using a 5× dry objective (HC PL Fluotar 5×/0.15). A 405 nm laser line was used to excite DAPI. Reflection mode was used to reproduce titanium surface by directly placing the detector over a 488 nm laser line. Sequential optical sections of 2.5 μm were collected along the z-axis over the complete thickness of the sample (~1 mm). The obtained images were processed with Las X software (Leica Microsystems CMS GmbH, Mannheim, Germany).

### 4.16. Statistical Analyses

Data are presented as mean ± SEM of three different biological preparations, each in technical duplicate. Statistical analyses were performed using R software version 4.0.3 (R Core Team, Wien, Austria). Simple comparisons between two groups were performed using Mann-Whitney test or Wilcoxon matched paired test. Two-way ANOVA with Tukey’s post-hoc test was used to evaluate differences among the study conditions. For qRT-PCR, level of significance was set at Log_2_(FC) ≥ 1 or ≤ −1, and *p*-value ≤ 0.05 (*p*-value ≤ 0.1 accepted for weak significance only with Log_2_(FC) ≥ 1 or ≤ −1). For biochemical assays, the same significance burdens were used with FC ≥ 2 or ≤ 0.5.

## Data Availability

Data is contained within the article or supplementary material.

## Supplementary Materials

Supplementary materials can be found at https://www.mdpi.com/1422-0067/22/5/2379/s1.

## Abbreviations

MSCs	Mesenchymal Stem Cells
T-Ti	Trabecular Titanium
PEEK	Polyetheretherketone
SEM	Scanning electron microscope
ALP	Alkaline Phosphatase
qRT-PCR	quantitative Real Time Polymerase Chain Reaction
